# BATS Code Decoding Method Based on Left-Nullspace-Guided Rank-Completion Feedback

**DOI:** 10.3390/e28080929

**Published:** 2026-08-19

**Authors:** Juan Yang, Jingjing Lu, Jianbo Ji, Tao Wang

**Affiliations:** 1 The School of Artificial Intelligence, Guilin University of Aerospace Technology, Guilin 541004, China; yangjuan@guat.edu.cn (J.Y.); jijianbo@guat.edu.cn (J.J.); 2 Guangxi Key Laboratory of Automatic Detecting Technology and Instruments, Guilin University of Electronic Technology, Guilin 541004, China; 3 Guangxi Zhuang Autonomous Region Engineering Research Center for Artificial Intelligence and Aerospace Information Scenarios, School of Artificial Intelligence, Guilin University of Aerospace Technology, Guilin 541004, China; 4 University Engineering Research Center of Integrated Aerospace-Terrestrial Information and Control, Guangxi, School of Artificial Intelligence, Guilin University of Aerospace Technology, Guilin 541004, China; 5 National Key Laboratory of Wireless Communications (NKLWC), University of Electronic Science and Technology of China (UESTC), Chengdu 610054, China; 202211220615@std.uestc.edu.cn

**Keywords:** BATS codes, feedback-assisted decoding, BP decoding, rank completion, left nullspace

## Abstract

BP decoding of BATS codes may stop when no residual batch satisfies the full-row-rank condition. To resume BP decoding, this paper proposes an Important-Packet-Guided Left-Nullspace Rank-Completion (LNRC) method. LNRC first identifies the unrecovered source packet that connects to the largest number of undecoded batches, denotes it as the Important Packet, and uses it as a guidance packet to locate the undecoded batches containing it as repair candidates. For a selected batch with rank deficit one, the destination computes a nonzero left-null vector and selects a local repair coordinate that provides the missing independent direction. The destination sends the corresponding global source-packet index and finite-field coefficient through a reliable reverse feedback-control link, and the source returns the scaled repair packet through a reliable forward repair-data link. The associated completion column increases the target residual transfer-matrix rank by one and makes the batch BP-decodable. Thus, LNRC exploits the column-space structure of the target undecoded batch to select the repair coordinate, rather than selecting the Important Packet solely by the number of connected undecoded batches. Under equal encoding redundancy, simulations show that LNRC achieves a lower packet error rate (PER) compared with conventional Important Packet feedback, with average relative PER reductions of approximately 0.40–12.17% across the evaluated settings.

## 1. Introduction

Reliable data transmission in multi-hop erasure networks, wireless relay networks, and sensor networks is a fundamental problem in communication systems. Due to factors such as link noise, congestion, and fading, packets may be lost during transmission. Traditional retransmission mechanisms typically rely on feedback channels to ensure transmission reliability; however, in long-distance, multi-hop, or high-loss scenarios, frequent feedback may incur substantial delay and control overhead.

Fountain codes can achieve data transmission without relying on feedback. The source continuously generates coded packets until the destination can recover the original data. BP decoding can be applied to fountain codes, offering high decoding reliability and low decoding complexity for long block lengths. However, in general networks, if intermediate nodes perform only store-and-forward operations, the optimal network transmission rate cannot be achieved. Random linear network coding allows intermediate nodes to linearly recombine the received coded packets, thereby improving the achievable transmission rate in multi-hop networks [1,2].

BATS codes combine the advantages of fountain codes and random linear network coding [3,4], requiring no feedback while providing high network transmission rates and low encoding and decoding complexity. Their outer code randomly selects source packets in batches and generates linear combinations, while their inner code allows intermediate nodes to perform random linear recoding within the same batch. Because network coding operations are confined to individual batches, BATS codes retain the advantages of rateless coding and network coding while offering good implementability [5].

At the destination, BATS decoding amounts to solving a finite-field system of linear equations formed by the received batches. Gaussian elimination can recover the source packets when the global system is solvable, but direct global elimination is not suitable as a low-overhead decoding scheme. BP decoding instead identifies batches whose transfer matrices have full row rank, recovers their source packets, and substitutes those packets into neighboring batches, potentially triggering cascaded decoding [4]. Improved BP decoding methods have also been studied [6]. However, BP decoding stops when every residual batch has transfer-matrix rank smaller than its residual degree [7,8].

Existing studies address this stopping condition through decoding-algorithm optimization and feedback-assisted recovery. Feedback-assisted methods introduce additional coded packets or source packets, thereby adding effective constraints to the residual decoding graph [9,10]. Related BATS-code designs have also used intermediate feedback, unequal error protection, and expanding windows to protect important or high-priority packets [9,11,12]. Wang [13] introduced Important Packet feedback, and Yang et al. [14] further considered source packets omitted by the outer encoder and applied recoding protection to batches containing important information.

Conventional Important Packet feedback operates as follows. After BP decoding stops, for each unrecovered source packet, the number of distinct undecoded batches that contain it is computed. The source packet connected to the largest number of undecoded batches is denoted as the Important Packet, and the source returns it as a repair packet through the reliable forward repair-data link. The destination receives the repair packet, substitutes it into the neighboring residual batches, which reduces their residual degrees, and resumes BP decoding. This procedure is repeated until all source packets are recovered or the repair-packet redundancy is exhausted. However, the conventional Important Packet selection criterion does not explicitly exploit the column-space structure of a designated residual batch. For a residual batch with rank deficit one, whether the requested source packet makes the batch immediately decodable depends on its local coordinate relative to the column space of the residual transfer matrix. Conventional Important Packet feedback therefore does not provide a deterministic rank-completion guarantee.

For the *i*th residual batch, the rank deficit is defined as

$$\delta_{i}=d_{i}-\mathrm{rank}\left(S_{i}\right),$$

where $d_{i}$ is the number of unrecovered source packets and $S_{i}$ is the corresponding residual transfer matrix. When $\delta_{i}=1$, the batch is one independent column away from the BP decodability condition. The proposed LNRC method uses the Important Packet as a guidance packet to narrow the search for candidate batches, i.e., undecoded batches associated with the guidance packet that have $\delta_{i}=1$. The nonzero support of a left-null vector then determines the local repair coordinate and the corresponding requested source packet. The scaled repair packet supplies a column outside the existing column space, guaranteeing that the augmented residual transfer matrix becomes full row rank after successful delivery. The guidance packet and the requested source packet need not be identical.

The main contributions of this paper are as follows:

1. A two-stage repair-selection mechanism is developed for stopped BP decoding. The Important Packet is used as a guidance packet to narrow the candidate-batch search, while the nonzero support of the selected batch’s left-null vector determines the requested source packet.
2. For a selected local coordinate *j* with $w_{j}\neq 0$, LNRC constructs $r=\alpha e_{j}$ with $\alpha =w_{j}^{-1}$. Since $w^{T}r=1$, the augmented residual transfer matrix is guaranteed to become full row rank after successful delivery of the repair packet.
3. LNRC is compared with standard BP decoding and conventional Important Packet feedback under equal encoding redundancy, isolating the benefit of left-nullspace-certified repair-coordinate selection.

The rest of this paper is organized as follows. Section 2 reviews BATS encoding, transmission, and BP decoding and introduces the feedback-assisted communication model. Section 3 presents the LNRC principle, mathematical construction, illustrative example, and repair algorithm. Section 4 presents the simulation results and discussion. Section 5 concludes the paper.

## 2. BATS Codes and the BP Decoding Stop Condition

To facilitate the description of the proposed repair algorithm, this section first reviews the encoding and decoding algorithms of BATS codes. Consider a source block containing *K* source packets, where each source packet is a length-*T* vector over GF(256). For the *i*th batch, the encoder first samples the batch degree $d_{i}$ according to a predefined degree distribution, and then randomly selects $d_{i}$ source packets from the *K* source packets to form the matrix $B_{i}$. It then generates the inner-batch coding matrix $G_{i}$, and the *M* coded packets transmitted in the *i*th batch are produced as follows:

(1) $$X_{i}=B_{i}G_{i},$$

where $G_{i}$ is a $d_{i}\times M$ generator matrix, and *M* is the batch size.

**Figure 1:**
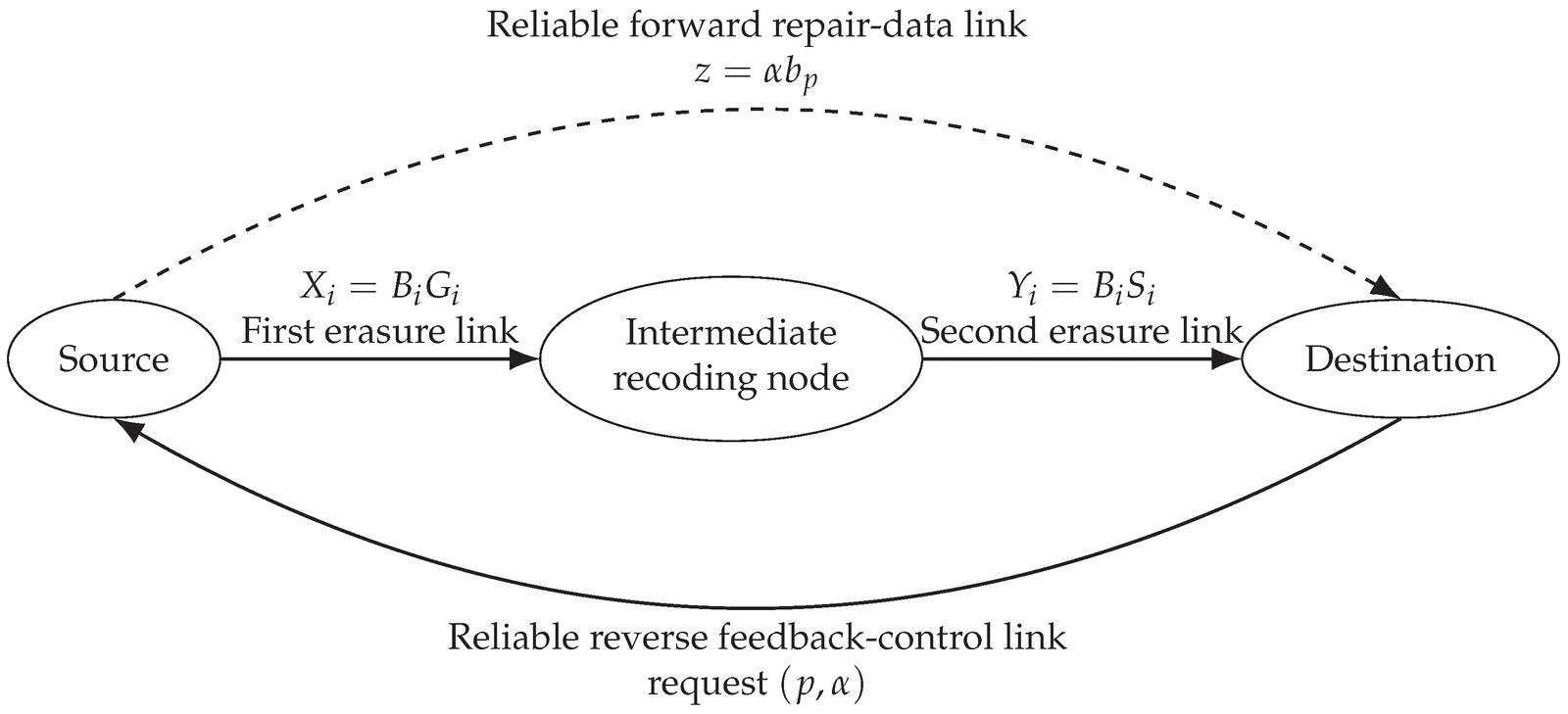
Two-hop BATS transmission and feedback-assisted repair model.

During network transmission, intermediate nodes perform random linear recoding only on coded packets within the same batch. Let $H_{i}$ denote the transfer matrix of the *i*th batch in the network; then the destination obtains

(2) $$Y_{i}=X_{i}H_{i}=B_{i}G_{i}H_{i}=B_{i}S_{i},$$

where $S_{i}=G_{i}H_{i}$ is the equivalent transfer matrix at the destination. As shown in Figure 1, the initial BATS packets traverse two erasure links through an intermediate recoding node. When BP decoding stops, the destination sends only the repair-request metadata (p,α) through a reliable reverse feedback-control link. The source then generates the repair packet

$$z=\alpha b_{p}$$

and transmits it through a reliable forward repair-data link. After receiving *z*, the destination locally constructs the completion column $r=\alpha e_{j}$ (different from the received repair packet *z*) and appends it to the selected residual batch.

At the destination, BATS decoding amounts to solving a finite-field system of linear equations formed by all received batches. BP decoding first computes the rank of the equivalent transfer matrix of each batch. For the *i*th batch, if

(3) $$\mathrm{rank}\left(S_{i}\right)=d_{i},$$

then the batch is decodable, and the destination can recover the $d_{i}$ source packets associated with it. The recovered source packets are then substituted into other undecoded batches, reducing their degrees and potentially creating new decodable batches. This process continues until no new decodable batch exists.

During BP decoding, recovered source packets are eliminated from the related batches. Let $d_{i}$ denote the number of unrecovered source packets in the *i*th residual batch, and continue to denote its residual transfer matrix by $S_{i}$. BP decoding stops when no residual batch satisfies $\mathrm{rank}\left(S_{i}\right)=d_{i}$. The residual rank deficit is then defined as follows:

(4) $$\delta_{i}=d_{i}-\mathrm{rank}\left(S_{i}\right).$$

Here, $d_{i}$ is the number of unrecovered source packets in the *i*th residual batch, and $S_{i}$ is its residual transfer matrix. A batch with $\delta_{i}=0$ already satisfies the BP decodability condition, whereas $\delta_{i}=1$ indicates that its residual transfer matrix lacks one linearly independent column for full row rank. The proposed LNRC method targets the latter case through single-symbol rank-completion repair.

## 3. Left-Nullspace-Based Rank-Completion Feedback Decoding Method

To improve recovery after BP decoding stops, this section develops LNRC, which combines residual-graph guidance with left-nullspace-certified rank completion. Conventional Important Packet feedback returns the Important Packet directly as a repair packet. LNRC instead uses the Important Packet only as a guidance packet to narrow the candidate-batch search. For a selected residual batch with rank deficit one, the nonzero support of a left-null vector determines a valid local repair coordinate, which is mapped to the requested source-packet index; thus, the requested source packet need not be the guidance packet. The source returns a finite-field-scaled repair packet, whose associated completion column lies outside the existing column space and raises the target residual transfer matrix to full row rank after successful delivery.

This section first derives the rank-completion condition and left-nullspace construction. It then establishes the relationship among the local repair coordinate, requested source-packet index, completion column, and transmitted repair packet. A three-dimensional example provides an intuitive interpretation, followed by a comparison with conventional Important Packet feedback and the complete LNRC repair algorithm.

### 3.1. Algorithm Principle: Rank Completion for a Deficit-One Residual Batch

Let the *i*th residual batch contain $d_{i}$ unrecovered source packets, collected in

(5) $$B_{i}=\left[b_{1},b_{2},\ldots ,b_{d_{i}}\right],$$

and let $S_{i}$ be its residual transfer matrix. The destination has

(6) $$Y_{i}=B_{i}S_{i}.$$

A batch is BP-decodable when $\mathrm{rank}\left(S_{i}\right)=d_{i}$. LNRC considers the deficit-one case

(7) $$\delta_{i}=d_{i}-\mathrm{rank}\left(S_{i}\right)=1,$$

which is equivalent to

(8) $$\mathrm{rank}\left(S_{i}\right)=d_{i}-1.$$

The batch therefore lacks one independent constraint for full row rank.

Suppose that an additional repair packet has coefficient column *r* satisfying

(9) $$r\notin \mathrm{Col}(S_{i}).$$

Then

(10) $$\mathrm{rank}\left(\left[S_{i},r\right]\right)=\mathrm{rank}\left(S_{i}\right)+1=d_{i}.$$

Let the corresponding repair packet be

(11) $$z=B_{i}r.$$

After receiving the repair packet, the destination has

(12) $$\left[Y_{i},z\right]=B_{i}\left[S_{i},r\right].$$

Because $\left[S_{i},r\right]$ has full row rank over GF(256), it has a right inverse ${\left[S_{i},r\right]}^{-R}$ satisfying

(13) $$\left[S_{i},r\right]{\left[S_{i},r\right]}^{-R}=I_{d_{i}}.$$

Hence,

(14) $$B_{i}=\left[Y_{i},z\right]{\left[S_{i},r\right]}^{-R}.$$

The residual batch selected for rank-completion repair is referred to as the target batch. LNRC directly repairs only residual batches with $\delta_{i}=1$. After the target batch is decoded, the newly recovered source packets are substituted into all neighboring residual batches. For a neighboring batch *k*, eliminating one recovered source packet changes its residual degree to $d_{k}^{\prime }=d_{k}-1$, while the rank of its residual transfer matrix decreases by at most one, i.e., $\mathrm{rank}\left(S_{k}^{\prime }\right)\geq \mathrm{rank}\left(S_{k}\right)-1$. Consequently, $\delta_{k}^{\prime }\leq \delta_{k}$, so substitution cannot increase the rank deficit and may reduce it. Repeated substitutions can therefore reduce a batch with $\delta_{k}>1$ to $\delta_{k}=1$, making it eligible for LNRC repair, or to $\delta_{k}=0$, making it BP-decodable. BP decoding is then resumed, and LNRC is invoked again only if BP decoding stops. Thus, although each LNRC operation directly completes only one deficit-one batch, the resulting decoding cascade can indirectly facilitate the recovery of batches with larger initial deficits.

### 3.2. Left-Nullspace-Guided Completion Column Construction

For a target residual batch with $\mathrm{rank}\left(S_{i}\right)=d_{i}-1$, there exists a nonzero left-null vector *w* such that

(15) $$w^{T}S_{i}=0^{T}.$$

Every column $x\in \mathrm{Col}(S_{i})$ therefore satisfies

(16) $$w^{T}x=0.$$

Because both $\mathrm{Col}(S_{i})$ and the null hyperplane $\left\{x:w^{T}x=0\right\}$ have dimension $d_{i}-1$, they are equal. Consequently,

(17) $$r\notin \mathrm{Col}\left(S_{i}\right)\;⟺\;w^{T}r\neq 0.$$

Here, *j* denotes a local repair coordinate, namely, a source-packet position within the selected target batch; it is not the index of the guidance packet. After computing *w*, the destination forms the nonzero support

(18) $$J=\{j:w_{j}\neq 0\}.$$

Because w≠0, the set J is nonempty. For any j∈J, the nonzero finite-field scalar $w_{j}$ has a multiplicative inverse. The destination sets

(19) $$\alpha =w_{j}^{-1},\;r=\alpha e_{j},$$

where $e_{j}$ is the *j*th standard basis vector. It follows that

(20) $$w^{T}r=w^{T}\left(\alpha e_{j}\right)=\alpha w_{j}=w_{j}^{-1}w_{j}=1\neq 0.$$

Thus, *r* lies outside the existing column space and

(21) $$\mathrm{rank}\left(\left[S_{i},r\right]\right)=\mathrm{rank}\left(S_{i}\right)+1=d_{i}.$$

The choice $\alpha =w_{j}^{-1}$ normalizes $w^{T}r$ to one; any nonzero α would provide the same rank increase when $w_{j}\neq 0$.

### 3.3. Distinction Between Completion Column *r* and Transmitted Repair Packet *z*

The completion column constructed by the destination and the repair packet transmitted by the source are different objects. Let *j* denote the selected local repair coordinate and let

(22) $$p={\mathrm{contributors}}_{i}\left(j\right)$$

be the corresponding global source-packet index. The destination constructs

(23) $$r=\alpha e_{j},$$

whereas the source transmits

(24) $$z=B_{i}r=\alpha b_{p}.$$

Thus, the source does not transmit *w*, $e_{j}$, or *r*; it transmits the repair packet *z*. The destination sends the repair-request metadata (p,α) through the reliable reverse feedback-control link, receives *z* through the reliable forward repair-data link, and locally appends *r* to the residual transfer matrix and *z* to the received data matrix:

(25) $$\left[Y_{i},z\right]=B_{i}\left[S_{i},r\right].$$

Since α≠0, the packet $z=\alpha b_{p}$ is a scaled repair packet. The rank-completion property comes from the left-nullspace-certified choice of the local repair coordinate, not from a new physical repair-packet format.

### 3.4. Three-Dimensional Vector Example: From a Two-Dimensional Subspace to Three- Dimensional Space

The following example verifies the direct one-packet rank-completion guarantee for a selected deficit-one target batch. After the target batch is decoded, all recovered source packets are substituted into neighboring residual batches, which can initiate cascaded decoding as described in Section 3.1. Consider a residual batch containing three unrecovered source packets:

(26) $$B=[b_{1},b_{2},b_{3}].$$

Although the algorithm operates over GF(256), this example uses GF(2) for clarity, where addition and subtraction coincide. The rank-completion criterion $w^{T}r\neq 0$ and the construction $r=\alpha e_{j}$ apply in the same way over GF(256). Let

(27) $$s_{1}=\left[\begin{array}{c} 1\\ 0\\ 1 \end{array}\right],\;s_{2}=\left[\begin{array}{c} 0\\ 1\\ 1 \end{array}\right],\;S=\left[s_{1},s_{2}\right]=\left[\begin{array}{cc} 1 & 0\\ 0 & 1\\ 1 & 1 \end{array}\right].$$

The destination initially has

(28) $$Y=BS=\left[y_{1},y_{2}\right],\;y_{1}=b_{1}+b_{3},\;y_{2}=b_{2}+b_{3}.$$

Since rank(S)=2 and d=3, the rank deficit is δ=1. A nonzero left-null vector is

(29) $$w=\left[\begin{array}{c} 1\\ 1\\ 1 \end{array}\right],$$

because, over GF(2),

$$w^{T}S=\left[1,1,1\right]\left[\begin{array}{cc} 1 & 0\\ 0 & 1\\ 1 & 1 \end{array}\right]=\left[0,0\right].$$

All three components of *w* are nonzero, so J={1,2,3} and each coordinate is valid for this example. Selecting j=1 gives $\alpha =w_{1}^{-1}=1$ and

(30) $$r=\alpha e_{1}=\left[\begin{array}{c} 1\\ 0\\ 0 \end{array}\right].$$

The source therefore transmits the repair packet $z=Br=b_{1}$. Since $w^{T}r=1\neq 0$, the appended column lies outside Col(S), as also verified by

$$\left[S,r\right]=\left[\begin{array}{ccc} 1 & 0 & 1\\ 0 & 1 & 0\\ 1 & 1 & 0 \end{array}\right]\overset{R_{3}\leftarrow R_{3}+R_{1}+R_{2}}{\to }\left[\begin{array}{ccc} 1 & 0 & 1\\ 0 & 1 & 0\\ 0 & 0 & 1 \end{array}\right].$$

Thus, rank([S,r])=3. After receiving $z=b_{1}$, the destination recovers

(31) $$b_{1}=z,\;b_{3}=y_{1}-z,\;b_{2}=y_{2}-b_{3}=y_{2}-y_{1}+z.$$

The request for $b_{1}$ is therefore not an arbitrary retransmission. It is selected because the corresponding coordinate vector $e_{1}$ satisfies the left-nullspace test $w^{T}e_{1}\neq 0$, which certifies that the completion column is linearly independent of the existing column space. One repair packet consequently makes the residual transfer-matrix full row rank and allows all three source packets in the batch to be recovered.

The fact that all three coordinates are valid is specific to this residual transfer matrix. In general, LNRC selects *j* only from $J=\{j:w_{j}\neq 0\}$; a coordinate with $w_{j}=0$ cannot increase the rank of the target batch.

**Figure 2:**
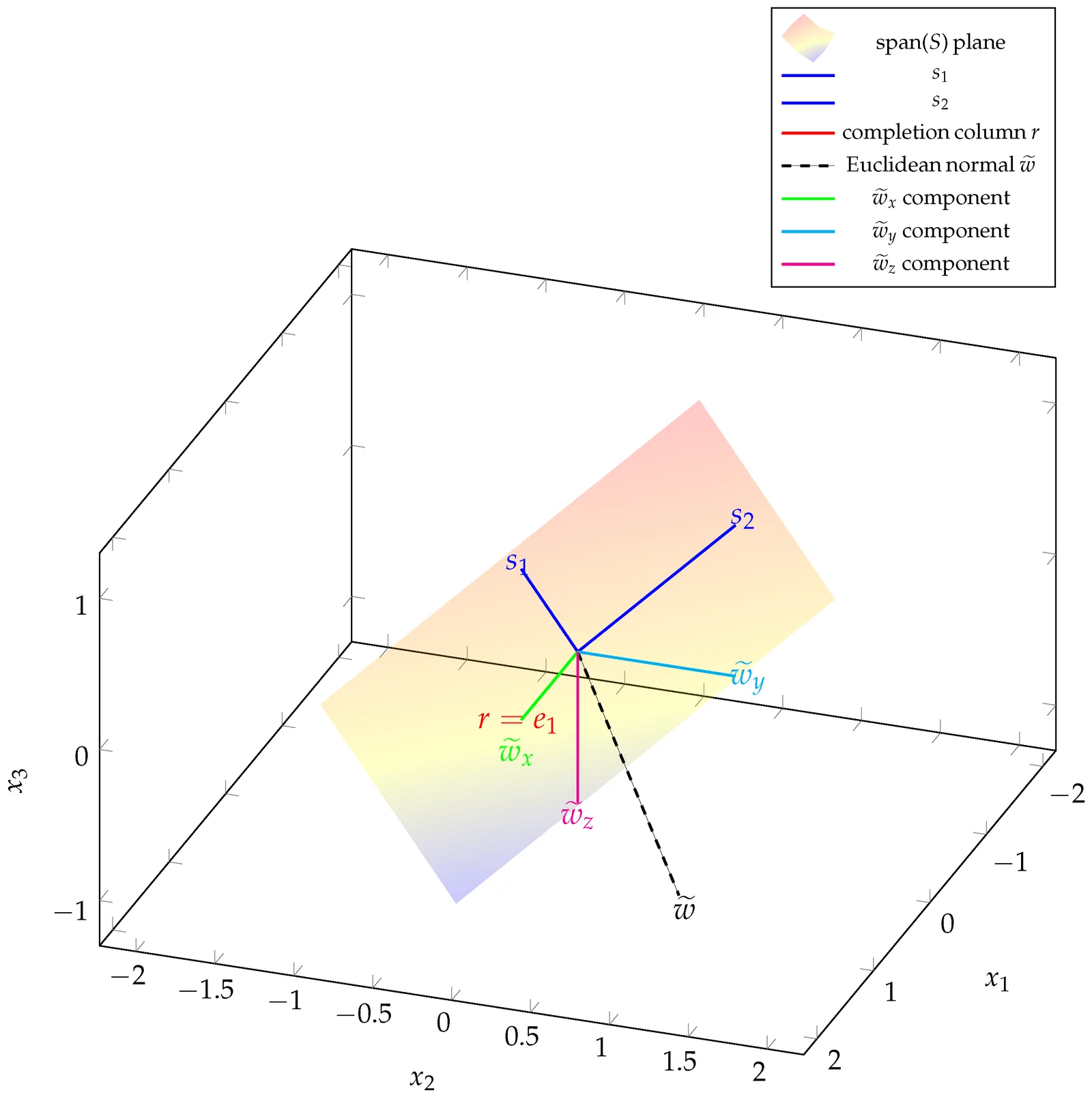
Geometric Illustration of LNRC Rank Completion (rank(S)=2).

Figure 2 provides a Euclidean analogy. Over the real coordinates used for visualization, the columns of *S* span the plane $x_{3}=x_{1}+x_{2}$, whereas $r=e_{1}$ lies outside that plane. The plotted Euclidean normal $\tilde{w}={\left[1,1,-1\right]}^{T}$ has the same nonzero-coordinate support as the finite-field left-null vector $w={\left[1,1,1\right]}^{T}$, since −1=1 in GF(2). The figure illustrates the coordinate-selection rule rather than a geometric embedding of $\mathrm{GF}{\left(2\right)}^{3}$.

### 3.5. Distinction from Conventional Important Packet Feedback

Conventional Important Packet feedback returns the Important Packet directly as a repair packet. Recovering the packet reduces the residual degrees of neighboring batches and may generate new decodable batches. For a specified deficit-one batch, however, immediate decodability depends on the packet’s local coordinate. If *w* is a nonzero left-null vector of the residual transfer matrix, the selected local coordinate *j* completes the rank only when

(32) $$w_{j}\neq 0.$$

Because conventional Important Packet feedback does not test this condition, it may resume decoding but does not certify the target batch. LNRC instead selects the local repair coordinate from $J=\{j:w_{j}\neq 0\}$. Both methods use repair packets; their distinction is whether the requested source packet is the Important Packet itself or is certified by the left-nullspace structure.

### 3.6. Important-Packet-Guided LNRC Algorithm

Algorithm 1 is invoked after BP decoding stops. It first identifies the Important Packet as the guidance packet *g* and uses *g* to narrow the candidate-batch search, then selects a target batch $i^{*}$ and uses the nonzero support of its left-null vector to determine the local repair coordinate *j* and the requested source-packet index *p*.
**Algorithm 1** Important-Packet-guided LNRC repair algorithm.**Require:** residual batches, recovered source packets, and repair-packet redundancy *F*
**Ensure:** updated recovered-source-packet set and repair-packet count
  1:Run BP decoding until it stops; f←0  2:**while** unrecovered source packets remain and f<F **do**  3:   Identify the Important Packet (the source packet connected to the largest number of undecoded batches) as the guidance packet *g*  4:   Find batches associated with *g* that satisfy $\delta_{i}=1$  5:   **if** no candidate batch exists **then**  6:       **break**  7:   **end if**  8:   Select a target batch $i^{*}$  9:   Compute a nonzero left-null vector *w* satisfying $w^{T}S_{i^{*}}=0^{T}$10:   Construct $J=\{j:w_{j}\neq 0\}$ and select j∈J11:   Map $p\leftarrow {\mathrm{contributors}}_{i^{*}}\left(j\right)$12:   Compute $\alpha \leftarrow w_{j}^{-1}$ and construct $r\leftarrow \alpha e_{j}$13:   Send repair request (p,α) through the reliable reverse feedback-control link14:   Receive repair packet $z\leftarrow \alpha b_{p}$ through the reliable forward repair-data link15:   Append *r* and *z* to target batch $i^{*}$16:   Decode the target batch17:   Substitute the recovered source packets into all neighboring residual batches and update their residual degrees and transfer matrices18:   f←f+119:   Restart BP decoding and run it until it stops20:**end while**21:Output recovered source packets


The guidance packet *g* is used only to narrow the candidate-batch search. After selecting a target batch $i^{*}$, LNRC chooses a local repair coordinate *j* from the nonzero support of its left-null vector and maps *j* to the requested source-packet index *p*. Therefore, the requested source packet *p* need not be the guidance packet *g*. The current implementation searches only undecoded batches associated with the guidance packet (the Important Packet). If none of these batches has $\delta_{i}=1$, the repair procedure terminates even though a repairable deficit-one batch may exist elsewhere in the residual graph. This coverage limitation could be addressed by searching all residual batches or by examining guidance packets in descending order of the number of associated undecoded batches.

The additional computation of LNRC occurs only at the destination during left-null-vector computation. Each feedback operation computes one left-null vector for one target residual batch and selects a repair coordinate from its nonzero support. The computation is determined by the residual transfer matrix of a single batch and requires only a small number of finite-field additions and multiplications.

## 4. Simulation Results and Discussion

### 4.1. Simulation Setup

The PER of BP, Important Packet feedback, and LNRC were evaluated by Monte Carlo simulation over GF(256). The batch size was M=16, the source-packet vector length was T=2, and each data point was averaged over 300 independent runs. The two initial BATS transmission links in Figure 1 have the same erasure probability *e*. Repair requests used the reliable reverse feedback-control link, and repair packets used the reliable forward repair-data link.

The asymptotic-degree-distribution cases used (e,K)=(0.1,10,000), (0.1,15,000), and (0.2,10,000). Two finite-length cases used K=512 with e=0.1 and e=0.2; their degree distributions were generated by a finite-length optimization program. Each delivered repair packet contributed one packet to the redundancy calculation, whereas the repair-request bits on the reverse feedback-control link were excluded. Important Packet feedback and LNRC used the same repair-packet redundancy and the same model for the reliable reverse feedback-control link and reliable forward repair-data link.

### 4.2. Comparison Methods and Evaluation Metrics

The simulations compared three methods:

- **BP**: standard BP decoding without repair; the number of transmitting batches is adjusted to the nearest feasible value given by (34).
- **Important Packet**: after BP decoding stops, the Important Packet is returned as a repair packet through the reliable forward repair-data link [13].
- **LNRC**: the proposed left-nullspace-certified rank-completion method.

The evaluation metric was the packet error rate (PER). The horizontal axis used the redundancy ρ, defined as follows:

(33) $$\rho =\frac{n_{\mathrm{TxBatch}}\cdot M+\bar{F}}{K}-1,$$

where $\bar{F}$ is the average number of repair packets used by LNRC. In each trial, Important Packet feedback used the same repair-packet redundancy as LNRC. For BP, this average redundancy was converted to the nearest feasible integer number of transmitting batches,

(34) $$n_{\mathrm{TxBatch},\mathrm{eq}}=\mathrm{round}\;\left(n_{\mathrm{TxBatch}}+\frac{\bar{F}}{M}\right).$$

The three methods were therefore compared at the same encoding redundancy, up to the integer-batch rounding required by BP.

### 4.3. PER Simulation Results

**Figure 3:**
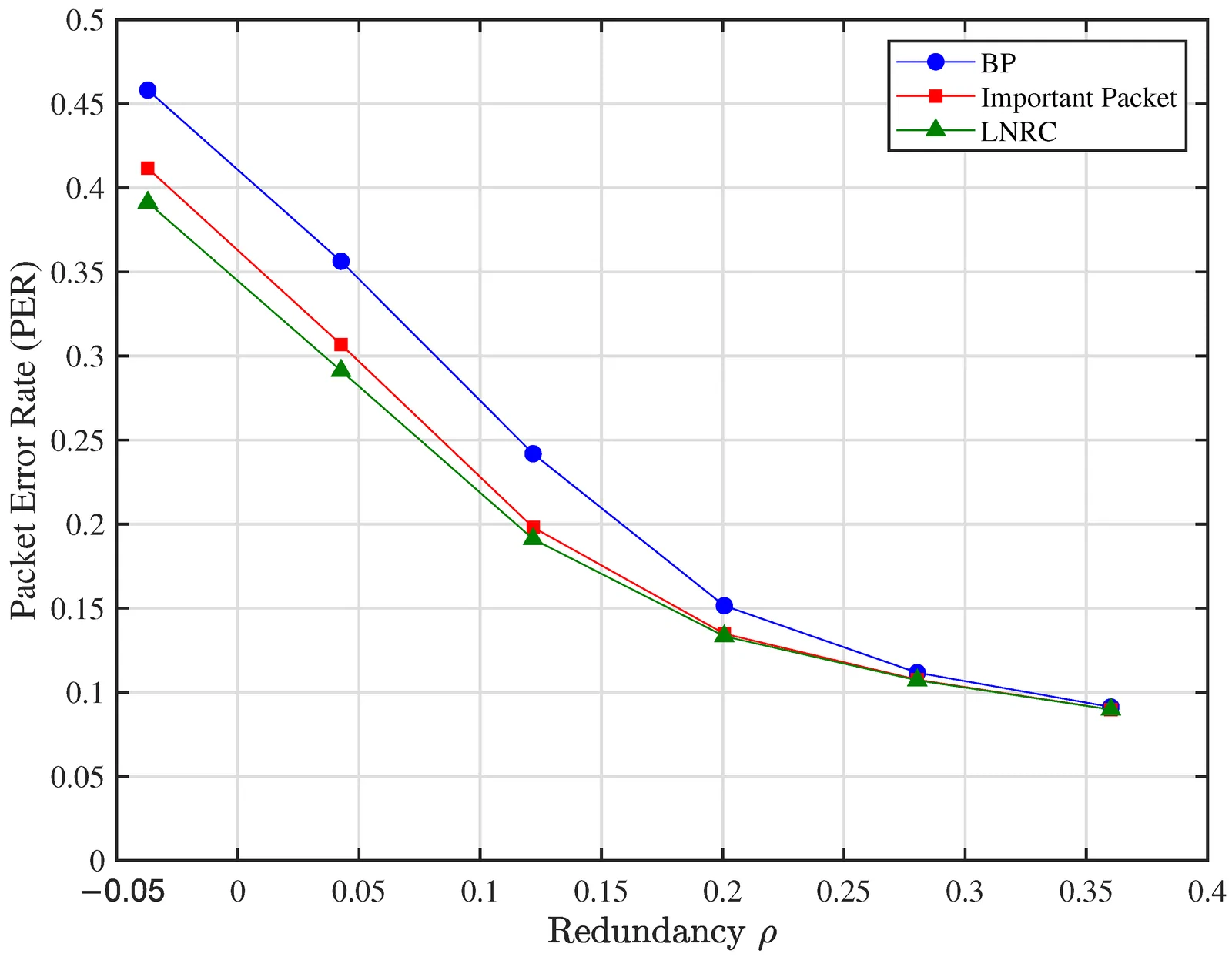
PER of BP, Important Packet feedback, and LNRC (e=0.1, K=10,000).

**Figure 4:**
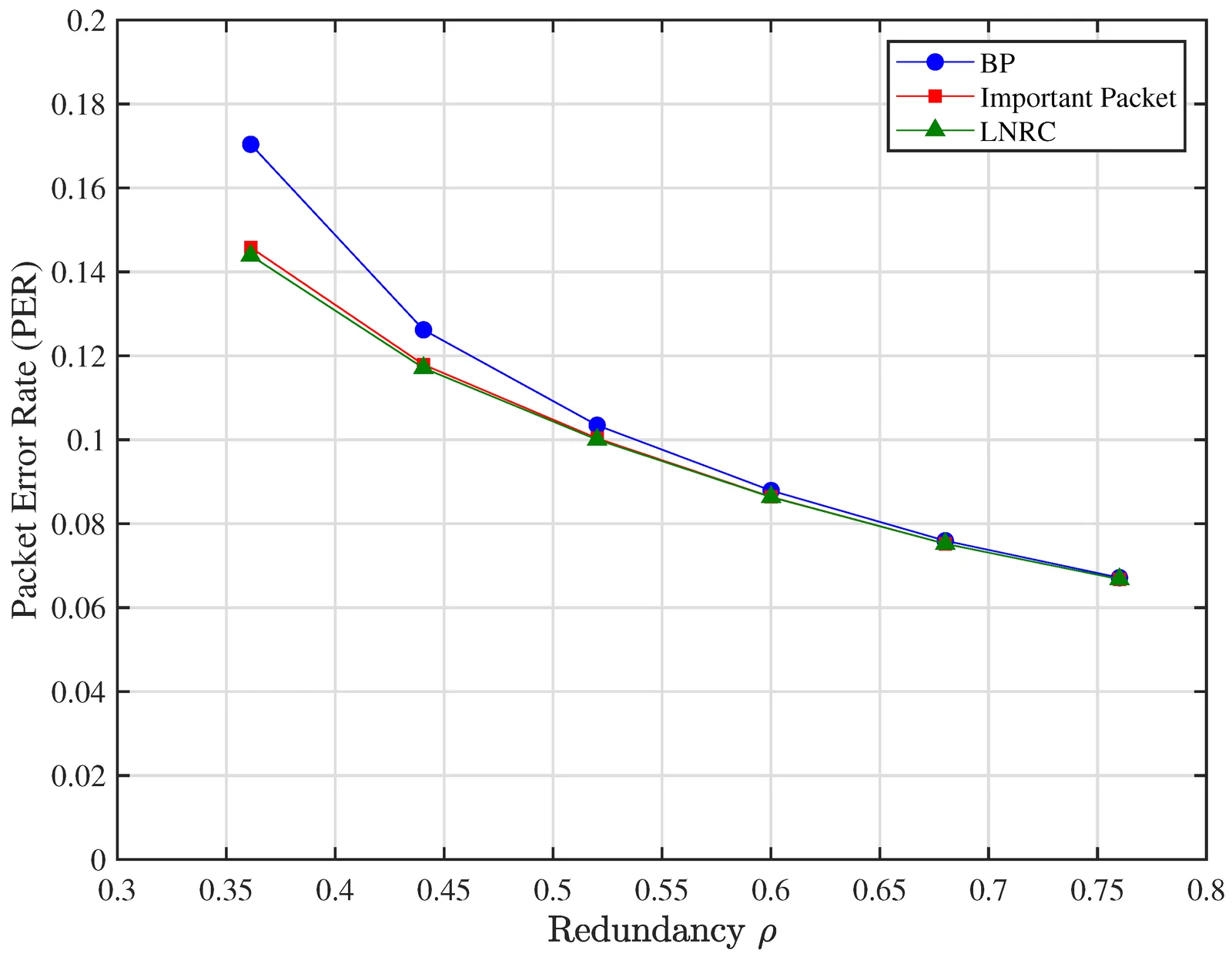
PER of BP, Important Packet feedback, and LNRC (e=0.2, K=10,000).

**Figure 5:**
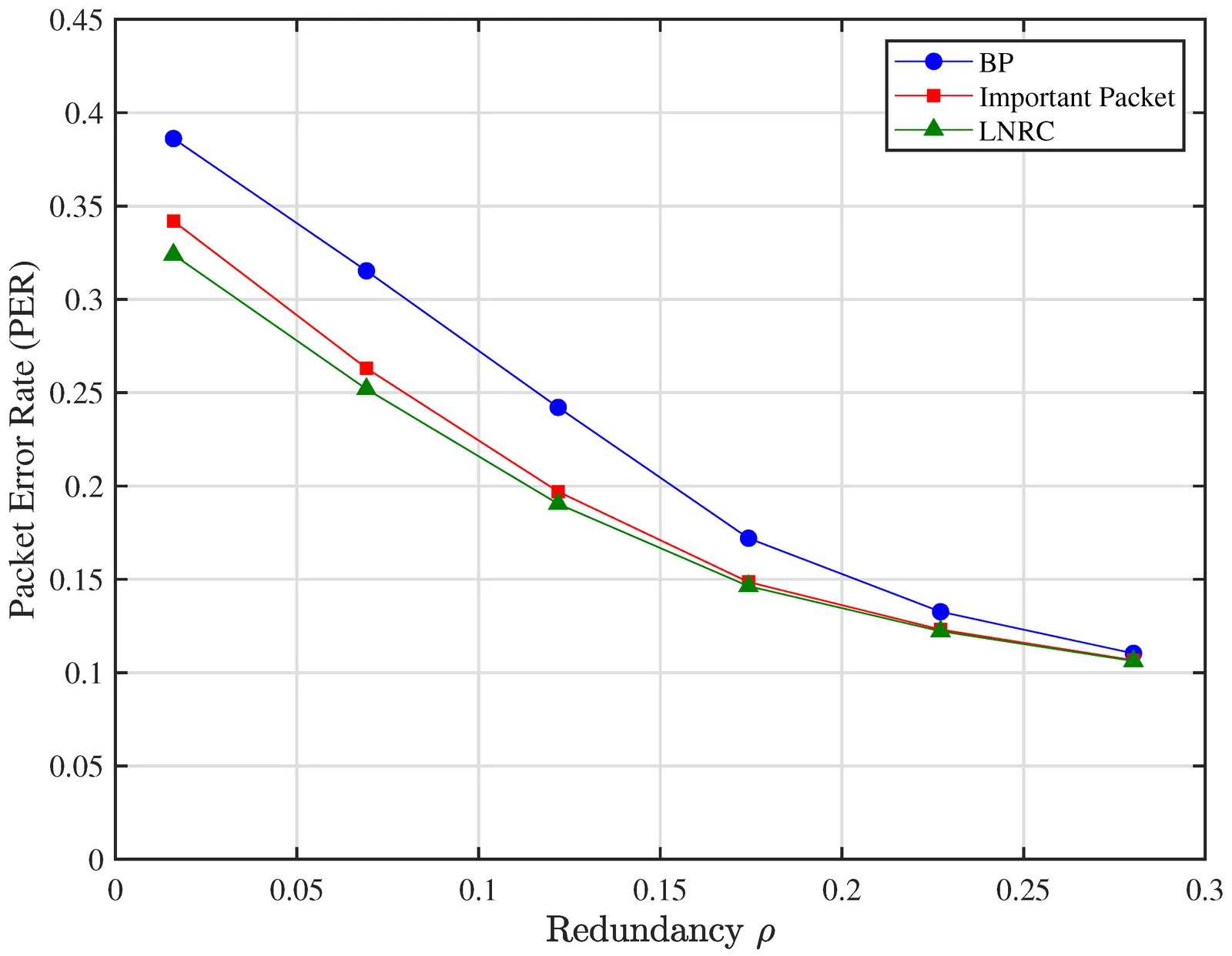
PER of BP, Important Packet feedback, and LNRC (e=0.1, K=15,000).

**Figure 6:**
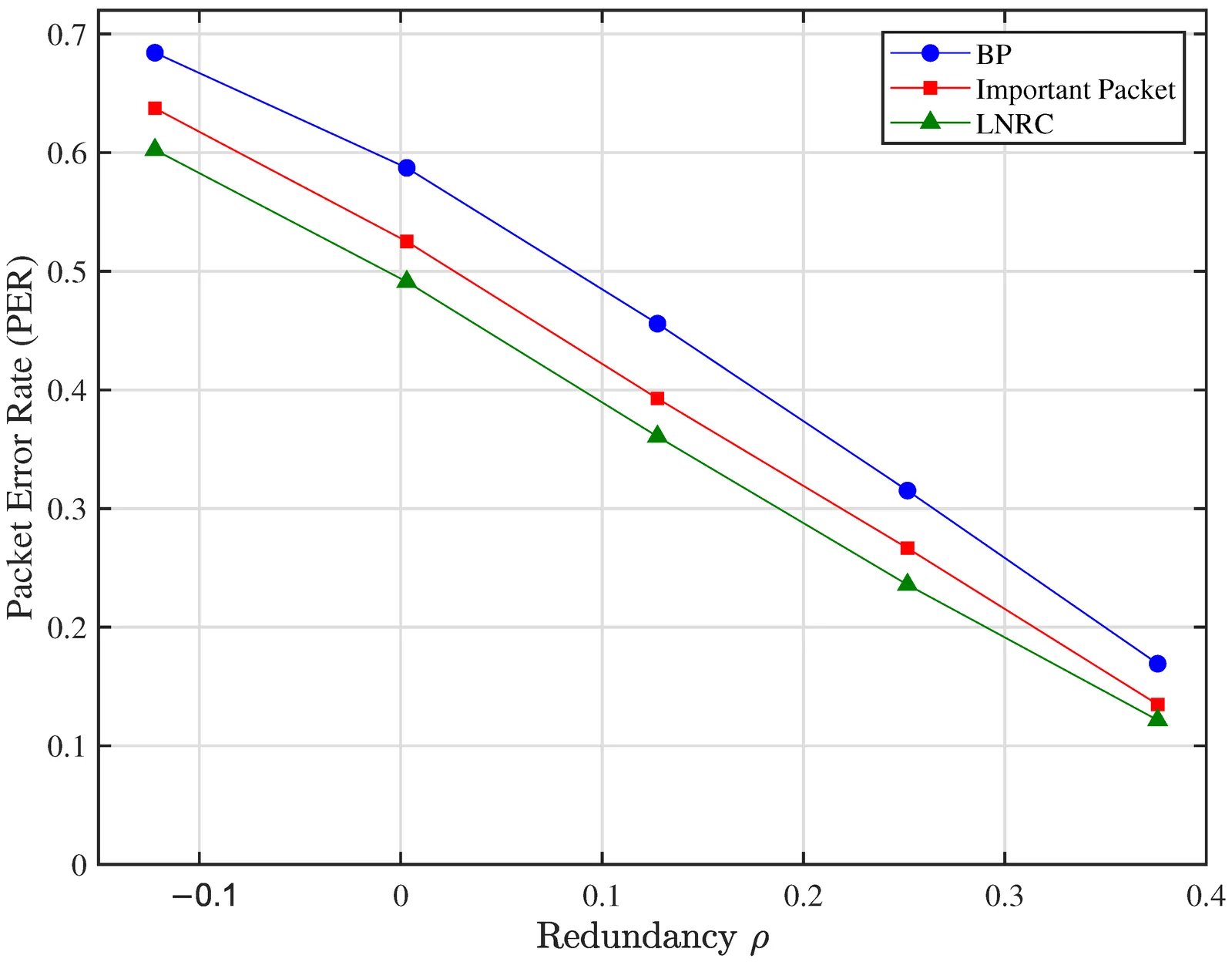
PER of BP, Important Packet feedback, and LNRC (e=0.1, K=512).

**Figure 7:**
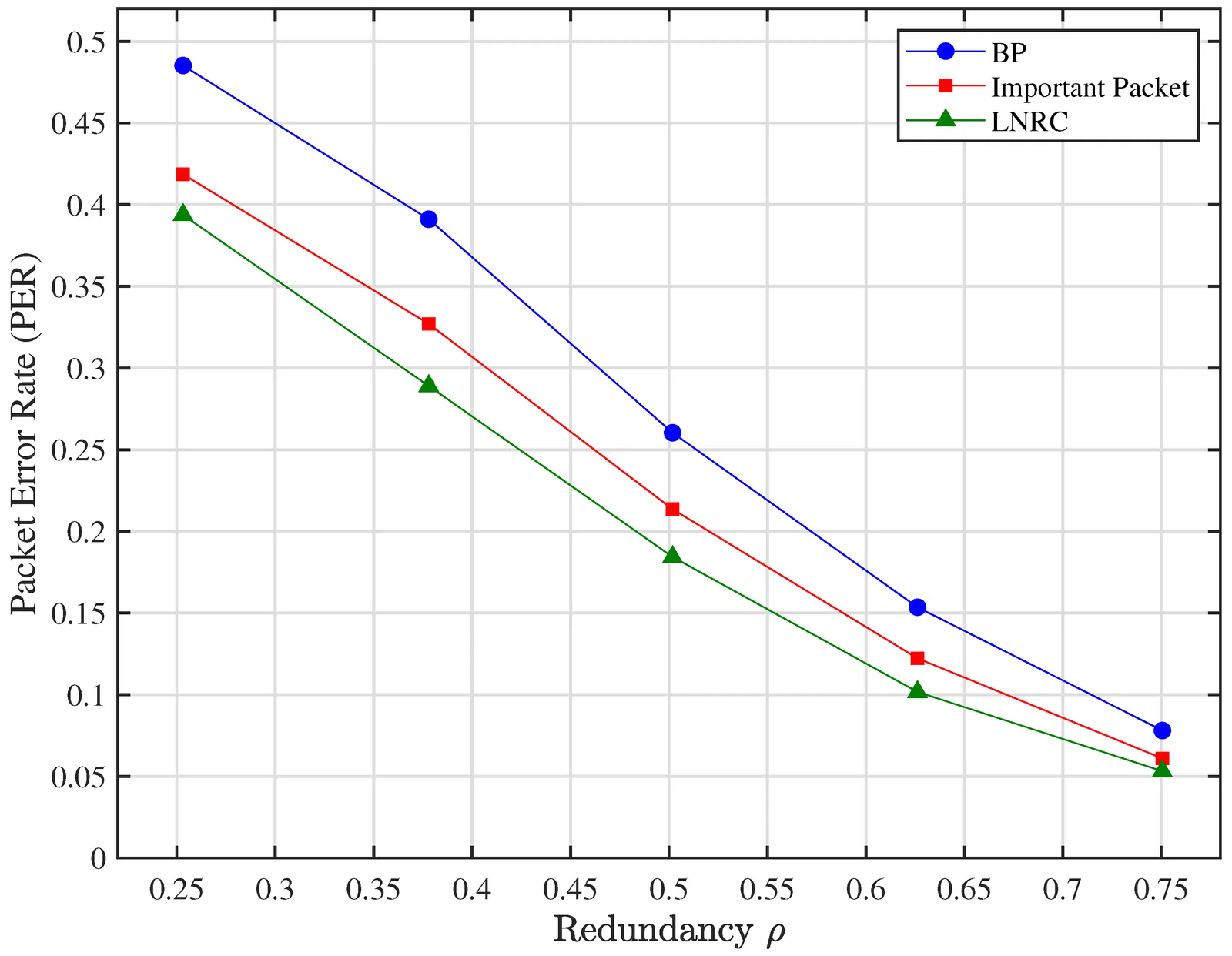
PER of BP, Important Packet feedback, and LNRC (e=0.2, K=512).

Figure 3, Figure 4, Figure 5, Figure 6 and Figure 7 compare the PER of BP, Important Packet feedback, and LNRC as a function of redundancy ρ.

For e=0.1, K=10,000, and $n_{\mathrm{Batch}}\in \left\{600,650,700,750,800,850\right\}$, LNRC achieves an average relative PER reduction of approximately 2.51% compared with conventional Important Packet feedback (Figure 3). The gain is largest at low redundancy and narrows as ρ increases, consistent with the stronger recovery capability of BP at higher redundancy.

For e=0.2, K=10,000, and $n_{\mathrm{Batch}}\in \left\{850,900,950,1000,1050,1100\right\}$, LNRC achieves an average relative PER reduction of approximately 0.40% compared with conventional Important Packet feedback (Figure 4). The limited gain is consistent with the strong recovery capability of BP in this setting and the relatively small number of residual deficit-one batches available for rank completion.

For e=0.1, K=15,000, and $n_{\mathrm{Batch}}\in \left\{950,1000,1050,1100,1150,1200\right\}$, LNRC achieves an average relative PER reduction of approximately 2.58% compared with conventional Important Packet feedback (Figure 5). The gain is concentrated primarily in the low-redundancy region, consistent with the stronger recovery capability of BP as ρ increases.

For the finite-length case with K=512, e=0.1, and $n_{\mathrm{Batch}}\in \left\{28,32,36,40,44\right\}$, LNRC achieves an average relative PER reduction of approximately 8.30% compared with conventional Important Packet feedback (Figure 6). The improvement is most pronounced in the low- and medium-redundancy regions, consistent with the ability of rank completion to resume BP decoding in finite-length settings.

For the finite-length case with K=512, e=0.2, and $n_{\mathrm{Batch}}\in \left\{40,44,48,52,56\right\}$, LNRC achieves an average relative PER reduction of approximately 12.17% compared with conventional Important Packet feedback, the largest improvement among the evaluated cases (Figure 7). Compared with Figure 6, the larger gain is consistent with a greater prevalence of repairable deficit-one batches in the residual graph under the higher-erasure setting.

### 4.4. Discussion of Results

Across the five evaluated cases, LNRC achieved lower PER than conventional Important Packet feedback under equal packet-equivalent redundancy. Important Packet feedback and LNRC used the same repair-packet redundancy and the same model for the reliable reverse feedback-control link and reliable forward repair-data link. Their performance difference therefore reflects the repair-selection rule: Important Packet feedback returned the Important Packet directly as the requested source packet, whereas LNRC used the Important Packet as a guidance packet and determines the local repair coordinate from the nonzero support of a left-null vector.

This gain is driven by cascaded decoding: a single LNRC repair directly completes one residual batch with $\delta_{i}=1$ and can then trigger cascaded BP decoding. The recovered source packets are substituted into neighboring residual batches, whose rank deficits cannot increase and may decrease; these batches may consequently become BP-decodable or enter the deficit-one candidate set in later repair rounds. Direct completion of a batch that remains at $\delta_{i}>1$ would require multiple independent repair columns.

The magnitude of this cascaded-decoding gain depends on the structure of the residual graph rather than on erasure probability alone. For K=10,000 and e=0.2, the average reduction was only 0.40%, whereas the two K=512 cases yielded 8.30% and 12.17%. The narrowing gap at higher redundancy is consistent with the reduced availability of repairable deficit-one batches after BP decoding. These results suggest that block length, degree distribution, redundancy, and the prevalence of batches with $\delta_{i}=1$ jointly affect the available LNRC gain.

However, these gains are obtained at the cost of additional computation during feedback-assisted repair. The dominant additional computation occurs at the destination only after BP decoding stops and a deficit-one target batch has been selected. In each feedback round, LNRC computes one nonzero left-null vector for the residual transfer matrix of that single target batch, constructs its nonzero support, and selects a repair coordinate. The source-side operation is limited to generating the requested scaled repair packet. Thus, LNRC does not introduce an additional global decoding procedure or modify the ordinary BP iterations. Table 1 quantifies this additional finite-field computation and compares it with the mean computation of one BP decoding trial at the same redundancies.

As shown in Table 1, the additional LNRC computation is substantially smaller than the mean BP decoding computation at every evaluated redundancy. The LNRC overhead decreases from approximately $4.0\times 10^{4}$ additions and $4.9\times 10^{4}$ multiplications at the lowest redundancy to approximately $1.2\times 10^{3}$ additions and $1.5\times 10^{3}$ multiplications at the highest redundancy, whereas one BP trial requires on the order of $10^{7}$ finite-field operations. Therefore, the performance gain of LNRC is achieved with a comparatively small destination-side computational cost.

The present study assumes a reliable reverse feedback-control link and a reliable forward repair-data link. If either link is unreliable, retransmission or acknowledgment mechanisms may be required. A global candidate search could also address the coverage limitation of the current guidance-based local search.

## 5. Conclusions

This paper proposes LNRC to resume BP decoding after it stops. LNRC uses the Important Packet as a guidance packet to narrow the candidate-batch search and determines the local repair coordinate from the nonzero support of a left-null vector of a selected deficit-one residual batch. For $\delta_{i}=1$, the completion column $r=\alpha e_{j}$ satisfies $w^{T}r\neq 0$ and raises the residual transfer matrix to full row rank after successful repair delivery. The source returns the corresponding scaled repair packet, so the contribution of LNRC lies in left-nullspace-certified repair-coordinate selection rather than a new repair-packet format. After the target batch is decoded, the recovered source packets are substituted into neighboring residual batches and may trigger further BP decoding.

Across the five evaluated cases, LNRC achieves lower PER than conventional Important Packet feedback under equal encoding redundancy, with average relative PER reductions ranging from approximately 0.40% to 12.17%. The magnitude of the gain depends jointly on block length, degree distribution, redundancy, and the prevalence of repairable deficit-one batches rather than on erasure probability alone. Simulation results further confirm that the additional computational overhead of LNRC is negligible compared with BP decoding.

The present method assumes a reliable reverse feedback-control link and a reliable forward repair-data link and uses a guidance-based local candidate-batch search. Future work will extend LNRC to unreliable reverse feedback-control and forward repair-data links, multi-column rank completion, and broader candidate searches that traverse lower-ranked guidance packets or examine the residual graph globally.

## Figures and Tables

**Table 1:** Comparison of LNRC’s additional computational overhead and mean BP computation at different redundancies.

Redundancy ρ	LNRC Add. GF Adds	LNRC Add. GF Muls	BP Mean GF Adds	BP Mean Equiv. GF Muls
−0.037069	40,318	49,391	$2.3771\times 10^{7}$	$2.6091\times 10^{7}$
0.042639	36,645	44,851	$2.7951\times 10^{7}$	$3.0778\times 10^{7}$
0.121831	25,216	30,890	$3.1409\times 10^{7}$	$3.4743\times 10^{7}$
0.200675	8949	11,002	$3.4465\times 10^{7}$	$3.8237\times 10^{7}$
0.280247	3097.8	3827.1	$3.6993\times 10^{7}$	$4.1123\times 10^{7}$
0.360098	1171.2	1453.2	$3.9546\times 10^{7}$	$4.4027\times 10^{7}$

## Data Availability

The data presented in this study are available on request from the corresponding author.

## Abbreviations

The following abbreviations are used in this manuscript:

BATS	BATched Sparse
BP	Belief Propagation
LNRC	Left-Nullspace Rank Completion
PER	Packet Error Rate
GF	Galois Field
